# The stability of a predator-prey system with linear mass-action functional response perturbed by white noise

**DOI:** 10.1186/s13662-016-0776-8

**Published:** 2016-02-18

**Authors:** Qiumei Zhang, Xiangdan Wen, Daqing Jiang, Zhenwen Liu

**Affiliations:** School of Science, Changchun University, Changchun, 130022 China; Department of Mathematics, Yanbian University, Yanji, 133002 China; College of Science, China University of Petroleum (East China), Qingdao, 266580 China; Nonlinear Analysis and Applied Mathematics (NAAM)-Research Group, King Abdulaziz University, Jeddah, Saudi Arabia; School of College of Basic Sciences, Changchun University of Technology, Changchun, 130021 China

**Keywords:** 92B05, 93E15, 60H10, 34E10, linear mass-action functional response, asymptotically stable, stochastically asymptotically stable

## Abstract

The present paper deals with the problem of an ecoepidemiological model with linear mass-action functional response perturbed by white noise. The essential mathematical features are analyzed with the help of the stochastic stability, its long time behavior around the equilibrium of deterministic ecoepidemiological model, and the stochastic asymptotic stability by Lyapunov analysis methods. Numerical simulations for a hypothetical set of parameter values are presented to illustrate the analytical findings.

## Introduction

In an ecosystem, species does not exist alone while it spreads the disease: it competes with the other species for space or food or is predated by other species. Therefore, it is essential to consider the effect of interacting species when we study the dynamical behaviors of epidemiological models. Recently, epidemiological dynamics have been extensively applied in population biology. Some researchers have made some achievements (see [1–11]).

The authors in [2] proposed and analyzed a predator-prey system in which some of the susceptible phytoplankton cells were infected by viral contamination and formed a new group (infected). The role of viral disease in recurrent phytoplankton blooms was discussed. They considered that the contact rate follows the law of proportional mixing rate. They did not take into account in their model that the infected phytoplankton cells become susceptible again. The author in [6] studied an SI or SIS model with disease spread among the prey when there is logistic growth of the predator and prey populations and when the predators eat infected prey only. They have not regarded that infected populations contribute to the susceptible population toward its carrying capacity. The authors in [9] modified the model equations of [2] and also the model of [6]. They assumed that the contact rate follows the law of mass action rate. A portion of infected phytoplankter was being recovered and became susceptible. The authors in [10] assumed that pelicans feed not only on infected fish but on susceptible fish also. Feeding on infected fish enhances the death rate of pelicans and is considered to contribute negative growth, whereas feeding on susceptible fish enhances their growth rate and is considered to contribute positive growth. In their model they did not consider that the portion of infected fish recovered and became susceptible. On the basis of this model, the authors in [11] studied and compared the dynamics of the proposed ecoepidemiological model to explore the crucial system parameters and their ranges in order to obtain different theoretical behaviors predicted from the interactions between susceptible prey, infected prey, and their predators. For linear mass-action functional response function, the ecoepidemiological model takes the following form:

1.1 $$ \left \{ \textstyle\begin{array}{@{}l} \frac{dS(t)}{dt}= rS(t)(1-\frac{S(t)+I(t)}{K})-\lambda S(t)I(t)-\alpha S(t)P(t),\\ \frac{dI(t)}{dt}= \lambda S(t)I(t)-\beta I(t)P(t)-\mu I(t),\\ \frac{dP(t)}{dt}= -\theta\beta I(t)P(t)-\delta P(t)+\theta\alpha S(t)P(t), \end{array}\displaystyle \right . $$

where $S(t)$, $I(t)$, $P(t)$ are the population densities of susceptible prey, infected prey, and predator, respectively, at time *t*, *K* is the carrying capacity, *r* is the growth rate of susceptible prey, *λ* is the force of infection, *θ* is the conversion efficiency, *α* and *β* are the attack rates on susceptible and infected prey, respectively, *μ* and *δ* are the death rates of the infected prey and predators, respectively.

The authors in [11] detail that system (1.1) has the following equilibria: $E_{0}=(0,0,0)$, $E_{1}=(K,0,0)$, $E_{2}=(\overline{S},\overline{I},0)=(\frac{\mu}{\lambda },\frac{r(1-\frac{\mu}{K\lambda})}{\frac{r}{K}+\lambda},0)$, $E_{3}=(\widetilde{S},0,\widetilde{P})=(\frac{\delta}{\theta\alpha },0,\frac{r}{\alpha}(1-\frac{\delta}{\theta\alpha K}))$, and $E_{*}=(S^{*},I^{*},P^{*})$, where

$$\begin{aligned} S^{*}=\frac{r+(\frac{r}{K}+\lambda)\frac{\delta}{\theta\beta}+\frac {\alpha\mu}{\beta}}{\frac{r}{K}+(\frac{r}{K}+2\lambda)\frac{\alpha }{\beta}},\qquad I^{*}=\frac{\theta\alpha S^{*}-\delta}{\theta\beta}, \qquad P^{*}=\frac{\lambda S^{*}-\mu}{\beta}. \end{aligned}$$

System (1.1) is unstable around $E_{0}$ for all parametric values, globally asymptotically stable around $E_{1}$ if $\lambda< \frac{\mu}{K} $ and $\alpha<\frac{\delta}{K\theta}$, globally asymptotically stable around $E_{2}$ if $\lambda> \frac{\mu}{K} $ and $\alpha<\frac{1}{\mu\theta}[\delta\lambda+\frac{r\beta\theta(\lambda K-\mu)}{r+\lambda K}]$, globally asymptotically stable around $E_{3}$ if $\lambda> \frac{\mu}{K} $ and $\alpha>\frac{\delta\lambda}{\mu \theta}$, and unstable around $E_{*}$ for all parametric values.

However, in this case, the effects due to environmental noise have been neglected. In fact, because of the existence of environmental noise, the parameters involved in system (1.1) are not absolute constants, and they fluctuate around some average values owing to continuous fluctuations in the environment. Therefore, the parameters in the model exhibit continuous oscillation around some average values but do not attain fixed values with the advancement of time. Consequently, the equilibrium population distribution fluctuates randomly around some average values. So many authors introduce stochastic perturbation into deterministic models to reveal the effect of environmental variability on the ecology and epidemiology system (see [12–16]). Keeping this in mind, we have modified the model (1.1) proposed by [11] and taken into account the effect of randomly fluctuating and stochastically perturbed force of infection *λ* in each equation of system (1.1):

$$\lambda\to\lambda+\sigma\dot{B}_{t}. $$

Consequently, $\lambda \,dt \to\lambda \,dt +\sigma \,dB_{t}$, where $B_{t}$ is a standard Brownian motion, $\sigma^{2}>0$ is the intensity of environmental white noise. Then system (1.1) becomes

1.2 $$ \left \{ \textstyle\begin{array}{@{}l} dS(t)= [rS(t)(1-\frac{S(t)+I(t)}{K})-\lambda S(t)I(t)-\alpha S(t)P(t)]\,dt-\sigma S(t)I(t)\,dB(t),\\ dI(t)= (\lambda S(t)I(t)-\beta I(t)P(t)-\mu I(t))\,dt+\sigma S(t)I(t)\,dB(t),\\ dP(t)= (-\theta\beta I(t)P(t)-\delta P(t)+\theta\alpha S(t)P(t))\,dt. \end{array}\displaystyle \right . $$

In this paper, we study the dynamics of the ecoepidemiological model with linear mass-action functional response perturbed by white noise to explore the crucial system parameters and their ranges in order to obtain different theoretical behaviors predicted from the interactions between susceptible prey, infected prey, and their predators.

This paper is organized as follows. The existence and uniqueness of a positive solution are given in Section 2. In Section 3, we show that the equilibrium $E_{0}$ of system (1.2) is stochastically unstable. In Section 4, we discuss that the equilibrium $E_{1}$ of system (1.2) is stochastically asymptotically stable in the large under some conditions and investigate the convergence rate of the solution. In Section 5, we study the fluctuations of system (1.2) about its equilibrium $E_{2}$ under some conditions. In Section 6, we carry out an analysis of stochastically asymptotically stability around the equilibrium $E_{3}$ of system (1.2). Numerical results are obtained by varying the parameters of the ecoepidemiological model in Section 7.

Throughout this paper, we let $(\Omega,\mathscr{F},\{\mathscr{F}_{t}\}_{t\geq0},\mathbb{P})$ be a complete probability space with filtration $\{\mathscr{F}_{t}\}_{t\geq0}$ satisfying the usual conditions (*i.e.*, it is increasing and right continuous with $\mathscr{F}_{0}$ containing all $\mathbb{P}$-null sets), and we let $B(t)$ be a scalar Brownian motion defined on the probability space.

## Existence and uniqueness of a positive solution

In this section, we show that there is a unique globally positive solution of system (1.2).

### Theorem 2.1

*There is a unique positive solution*$(S(t),I(t),P(t))$*of system* (1.2) *a*.*s*. *for any initial value*$(S(0),I(0),P(0))\in R^{3}_{+}$, *and*$S(0)+I(0)\leq K$.

### Proof

Obviously, the coefficients of equation (1.2) satisfy the local Lipschitz condition. Therefore, there is a unique local solution $(S(t),I(t),P(t))$ on $t \in[0,\tau_{e})$, where $\tau_{e}$ is the explosion time. Moreover, if $S(0)+I(0)\leq K$, then $S(t)+I(t)\leq K$ for $t \in[0,\tau_{e})$ a.s. In fact, note that

$$\begin{aligned} \frac{d(S+I)}{dt}&=rS \biggl(1-\frac{S+I}{K} \biggr)-\alpha SP-\beta IP- \mu I\\ &\leq\max \biggl\{ 0,r(S+I) \biggl(1-\frac{S+I}{K} \biggr) \biggr\} . \end{aligned}$$

Therefore,

$$S(t)+I(t) \leq\max \biggl\{ S(0)+I(0), \biggl[\frac{1}{K}+ \biggl( \frac{1}{S(0)+I(0)}-\frac {1}{K} \biggr)e^{-rt} \biggr]^{-1} \biggr\} \leq K. $$

Let $W(t)=S(t)+I(t)+\frac{1}{\theta}P(t)$. Then

$$\begin{aligned} \frac{dW(t)}{dt}+\eta W(t) &=rS \biggl(1-\frac{S+I}{K} \biggr)-2\beta IP- \mu I-\frac{\delta}{\theta}P+\eta \biggl(S+I+\frac{1}{\theta}P \biggr) \\ &\leq S \biggl[\eta+r \biggl(1-\frac{S}{K} \biggr) \biggr]-(\mu-\eta)I- \frac{\delta-\eta}{\theta}P \\ &=-\frac{r}{K} \biggl[S-\frac{K(r+\eta)}{2r} \biggr]^{2}+ \frac{K(r+\eta)^{2}}{4r}-(\mu -\eta)I-\frac{\delta-\eta}{\theta}P \\ &\leq\frac{K(r+\eta)^{2}}{4r} \end{aligned}$$

by choosing $\eta=\min\{\mu,\delta\}$ such that $\mu-\eta\geq0$ and $\delta-\eta\geq0$. Hence, by the comparison theorem we get

$$\begin{aligned} W(t)&\leq e^{-\eta t} \biggl[W(0)+\frac{K}{4r\eta}(\eta+r)^{2} \bigl(e^{\eta t}-1 \bigr) \biggr]\\ &\leq\max \biggl\{ W(0),\frac{K}{4r\eta}(\eta+r)^{2} \biggr\} \end{aligned}$$

and

$$\limsup_{t\rightarrow\infty}W(t)\leq\frac{K}{4r\eta}(\eta+r)^{2}:= \frac {B}{\eta} \biggl(B=\frac{K(\eta+r)^{2}}{4r} \biggr), $$

which is independent of the initial values.

Now, we are going to show that this solution is global, that is, that $\tau_{e}=\infty$ a.s. Let $k_{0}>0$ be sufficiently large so that $S(0)$, $I(0)$, and $P(0)$ all lie within the interval $[\frac {1}{k_{0}},k_{0}]$. For each integer $k \geq k_{0}$, define the stopping time

$$\tau_{k}=\inf \biggl\{ t\in[0,\tau_{e}):\min \bigl\{ S(t),I(t),P(t) \bigr\} \leq\frac{1}{k} \mbox{ or } \max \bigl\{ S(t),I(t),P(t) \bigr\} \geq k \biggr\} , $$

where we set $\inf\emptyset= \infty$ (as usual, ∅ denotes the empty set). Clearly, $\tau_{k}$ is increasing as $k \rightarrow \infty$. Set $\tau_{\infty}=\lim_{k \rightarrow\infty}\tau_{k}$, whence $\tau_{\infty}\leq\tau_{e}$ a.s. If we can show that $\tau _{\infty}=\infty$ a.s., then $\tau_{e}=\infty$ and $(S(0),I(0),P(0))\in R^{3}_{+}$ a.s. for all $t \geq0$. In other words, to complete the proof, all we need to show is that $\tau_{\infty}=\infty$ a.s. If this statement is false, then there is a pair of constants $T > 0$ and $\varepsilon\in(0,1)$ such that

$$\mathbb{P}\{\tau_{\infty} \leq T \}> \varepsilon. $$

Hence, there is an integer $k_{1} \geq k_{0}$ such that

2.1 $$ \mathbb{P}\{\tau_{k} \leq T \}\geq\varepsilon \quad \mbox{for all } k \geq k_{1}. $$

Define the $C^{2}$-function $V:R^{3}_{+} \rightarrow\overline{R}_{+}$ by

$$V(S,I,P)=S-1-\log S+I-1-\log I+\frac{1}{\theta}(P-1-\log P). $$

The nonnegativity of this function can be seen from the inequality $u-l-l\log\frac{u}{l}\geq0 $ ($l>0$) for all $u>0$. Using Itô’s formula, we get

$$\begin{aligned} d V ={}& \biggl[(S-1) \biggl(r \biggl(1-\frac{S+I}{K} \biggr)-\lambda I- \alpha P \biggr)+\frac{\sigma ^{2}I^{2}}{2} \biggr]\,dt-\sigma I(S-1)\,dB(t) \\ &{}+ \biggl[(I-1) (\lambda S-\beta P-\mu)+\frac{\sigma^{2}S^{2}}{2} \biggr]\,dt+\sigma S(I-1)\,dB(t)\\ &{}+\frac{1}{\theta}(P-1) (-\theta\beta I-\delta+\theta\alpha S)\,dt \\ :={}&LV \,dt+\sigma(I-S)\,dB(t), \end{aligned}$$

where

$$\begin{aligned} LV={}&\mu+\frac{\delta}{\theta}-r+ \biggl(r+\frac{r}{K}-\lambda-\alpha \biggr)S+ \biggl(\frac {r}{K}+\lambda+\beta-\mu \biggr)I+ \biggl(\alpha+ \beta- \frac{\delta}{\theta} \biggr)P \\ &{}-\frac{r}{K}S^{2}-\frac{r}{K}SI- 2 \beta IP+ \frac{\sigma ^{2}}{2} \bigl(S^{2}+I^{2} \bigr) \\ \leq{}&\mu+\frac{\delta}{\theta}+ \biggl(r+\frac{r}{K} \biggr)S+ \biggl( \frac{r}{K}+\lambda +\beta \biggr)I+(\alpha+\beta)P+\frac{\sigma^{2}}{2} \bigl(S^{2}+I^{2} \bigr) \\ \leq{}&\mu+\frac{\delta}{\theta}+\max \biggl\{ r+\frac{r}{K}, \frac{r}{K}+\lambda +\beta,\theta(\alpha+\beta) \biggr\} \frac{B}{\eta}+K^{2}\sigma^{2}. \end{aligned}$$

By a similar proof as in Li and Mao [16], Theorem 2.1, we can obtain the desired assertion; see Appendix 2. □

### Remark 2.1

From this theorem we know that the region

$$\Gamma= \biggl\{ (S,I,P)\in R^{3}_{+}: S+I\leq K, S+I+ \frac{1}{\theta}P\leq\frac {B}{\eta} \biggr\} $$

is a positively invariant set of system (1.2), where *B* and *η* are determined in the proof of Theorem 2.1. From now on we always assume that the initial value $(S(0),I(0),P(0))\in\Gamma$.

## Stochastic instability around the equilibrium $E_{0}=(0,0,0)$

System (1.1) is unstable around $E_{0}$ for all parametric values. It is obvious that $E_{0}$ is still an equilibrium of system (1.2). In this section, we show that the equilibrium $E_{0}$ of system (1.2) is stochastically unstable.

### Theorem 3.1

*Let*$(S(t),I(t),P(t))$*be the solution of system* (1.2) *with initial value*$(S(0),I(0), P(0))\in\Gamma$. *Then the equilibrium*$E_{0}=(0,0,0)$*of system* (1.2) *is stochastically unstable*.

### Proof

If not, there must be $\Omega_{0}$ and $T_{0}>0$ such that $\mathbb{P}\{ \Omega_{0}\}>0$ and $S(t)\leq\frac{K}{4}$, $I(t)\leq\frac {Kr}{2(2r+2K\lambda+K^{2}\sigma^{2})}$, and $P(t)\leq\frac{r}{4\alpha}$ for $t \geq T_{0}$, $\omega\in\Omega_{0}$. Hence,

$$\begin{aligned} d\log S&= \biggl[r-\frac{r}{K}S- \biggl(\frac{r}{K}+\lambda \biggr)I- \alpha P-\frac{\sigma ^{2}}{2}I^{2} \biggr]\,dt-\sigma I\,dB(t) \\ &\geq \biggl[r-\frac{r}{K}S- \biggl(\frac{r}{K}+\lambda+ \frac{K\sigma ^{2}}{2} \biggr)I-\alpha P \biggr]\,dt-\sigma I\,dB(t) \\ &\geq\frac{r}{4}\,dt-\sigma I\,dB(t). \end{aligned}$$

Then

$$\log S(t)-\log S(T_{0})\geq\frac{r}{4}(t-T_{0})- \sigma \int ^{t}_{T_{0}}I(s)\,dB(s). $$

Let $M(t)=\int^{t}_{T_{0}}I(s)\,dB(s)$, which is a real-valued continuous local martingale, $M(T_{0})=0$, and

$$\limsup_{t\rightarrow\infty}\frac{\langle M,M\rangle_{t}}{t}=\limsup_{t\rightarrow\infty} \frac{\int^{t}_{T_{0}}I^{2}(s)\,ds}{t}\leq K^{2}< \infty \quad\mbox{a.s. } $$

Then by the strong law of large numbers we have

$$\lim_{t\rightarrow\infty}\frac{M(t)}{t}=\lim_{t\rightarrow\infty } \frac{\int^{t}_{T_{0}}I(s)\,dB(s)}{t}=0 \quad\mbox{a.s. } $$

Therefore,

$$\liminf_{t\rightarrow\infty}\frac{\log S(t)}{t}\geq\frac{r}{4}, $$

which is a contradiction, and the proof of this theorem is completed. □

## Global asymptotic stability around the equilibrium $E_{1}=(K,0,0)$

System (1.1) is globally asymptotically stable around $E_{1}$ if $\lambda< \frac{\mu}{K} $ and $\alpha<\frac{\delta}{K\theta}$. It is obvious that $E_{1}$ is still an equilibrium of system (1.2). In this section, we first show that it is stochastically asymptotically stable in the large under some conditions. Then we investigate the convergence rate of the solution.

### Theorem 4.1

*Let*$(S(t),I(t),P(t))$*be the solution of system* (1.2) *with initial value*$(S(0),I(0), P(0))\in\Gamma$. *If*$K \lambda<\mu-\frac{\lambda K^{3}\sigma^{2}}{2(r+K\lambda)}$*and*$\alpha<\frac{\delta}{K\theta}$, *then the equilibrium*$E_{1}=(K,0,0)$*of system* (1.2) *is stochastically asymptotically stable in the large*.

### Proof

Define the function $V:R^{3}\rightarrow R_{+}$ by

$$V(S,I,P)=S-K-K\log\frac{S}{K}+\frac{\frac{r}{K}+\lambda}{\lambda }I+\frac{1}{\theta}P. $$

Let *L* be the generating operator of system (1.2). Then

$$\begin{aligned} LV={}&(S-K) \biggl[r \biggl(1-\frac{S+I}{K} \biggr)-\lambda I-\alpha P \biggr]+ \frac{K\sigma ^{2}}{2}I^{2}+\frac{\frac{r}{K}+\lambda}{\lambda}(\lambda SI-\beta IP- \mu I) \\ &{}+\frac{1}{\theta}(-\theta\beta IP-\delta P+\theta\alpha SP) \\ ={}&-\frac{r}{K}(S-K)^{2}+ \biggl[K \biggl(\frac{r}{K}+ \lambda \biggr)-\frac{\frac {r}{K}+\lambda}{\lambda}\mu \biggr]I- \biggl(\frac{\frac{r}{K}+\lambda}{\lambda }+1 \biggr) \beta IP-\frac{\delta}{\theta}P \\ &{}+\frac{K\sigma ^{2}}{2}I^{2}+\alpha SP \\ ={}&-\frac{r}{K}(S-K)^{2}+ \biggl[K \biggl(\frac{r}{K}+ \lambda \biggr)-\frac{\frac {r}{K}+\lambda}{\lambda}\mu+\frac{K\sigma^{2}}{2}I \biggr]I\\ &{}+ \biggl(\alpha S- \frac {\delta}{\theta}- \biggl(\frac{\frac{r}{K}+\lambda}{\lambda}+1 \biggr)\beta I \biggr)P \\ \leq{}& -\frac{r}{K}(S-K)^{2}+ \biggl[K \biggl( \frac{r}{K}+\lambda \biggr)-\frac{\frac {r}{K}+\lambda}{\lambda}\mu+\frac{K^{2}\sigma^{2}}{2} \biggr]I+ \biggl(\alpha K-\frac {\delta}{\theta} \biggr)P, \end{aligned}$$

which is negative-definite according to $K(\frac{r}{K}+\lambda)-\frac{\frac{r}{K}+\lambda}{\lambda}\mu+\frac {K^{2}\sigma^{2}}{2}<0$ and $\alpha K-\frac{\delta}{\theta}< 0$, that is, $K\lambda-\mu+\frac{\lambda K^{3} \sigma^{2}}{2(r+K\lambda)}<0$ and $\alpha<\frac{\delta}{K\theta}$. Therefore, by Lemma A.2 (Mao [17]) the equilibrium $E_{1}=(K,0,0)$ of system (1.2) is stochastically asymptotically stable in the large. □

In the remainder of this section, we compute the convergence rate of $I(t)$, $P(t)$, and $S(t)$.

### Theorem 4.2

*Let*$(S(t),I(t),P(t))$*be the solution of system* (1.2) *with initial value*$(S(0),I(0), P(0))\in\Gamma$. *Assume that*

- $\sigma^{2}>\max\{\frac{\lambda}{K},\frac{\lambda^{2}}{2\mu}\}$, *or*
- $\max\{0,\frac{2(\lambda K-\mu)}{K^{2}}\}<\sigma^{2}\leq\frac {\lambda}{K}$, *or*
- $\alpha<\frac{\delta}{K\theta}$.

*Then*

$$\begin{aligned} &\limsup_{t\rightarrow\infty}\frac{\log I(t)}{t}\leq\frac{\lambda ^{2}}{2\sigma^{2}}-\mu< 0 \quad\textit{a.s. if }(\mathrm{a})\textit{ holds}; \\ &\limsup_{t\rightarrow\infty}\frac{\log I(t)}{t}\leq\lambda K-\mu - \frac{\sigma^{2}K^{2}}{2}< 0\quad\textit{a.s. if }(\mathrm{b})\textit{ holds}; \\ &\limsup_{t\rightarrow\infty}\frac{\log P(t)}{t}\leq-( \delta-\theta \alpha K)< 0 \quad\textit{a.s. if }(\mathrm{c})\textit{ holds}. \end{aligned}$$

*Moreover*,

$$\begin{aligned} \lim_{t\rightarrow\infty}\frac{1}{t} \int^{t}_{0}S(s)\,ds=K \quad \textit{a.s.} \end{aligned}$$

### Proof

By Itô’s formula we have

$$ d\log I= \biggl(\lambda S-\beta P-\mu-\frac{ \sigma^{2}}{2}S^{2} \biggr)\,dt+ \sigma S\,dB(t)\leq \biggl(\lambda S-\mu-\frac{ \sigma^{2}}{2}S^{2} \biggr)\,dt+ \sigma S\,dB(t). $$

Let

$$ f(S):=\lambda S-\mu-\frac{ \sigma^{2}}{2}S^{2}, \quad s\in(0,K]. $$

We will analyze the following two cases.

(i) $\frac{\lambda}{\sigma^{2}}< K$. Then we have

$$ f(S)\leq f \biggl(\frac{\lambda}{\sigma^{2}} \biggr)=\frac{\lambda^{2}}{2\sigma ^{2}}-\mu. $$

Therefore,

$$ d\log I\leq \biggl(\frac{\lambda^{2}}{2\sigma^{2}}-\mu \biggr)\,dt+\sigma S\,dB(t) $$

and

4.1 $$ \frac{\log I(t)}{t}\leq\frac{\log I(0)}{t}+ \biggl( \frac{\lambda^{2}}{2\sigma^{2}}-\mu \biggr)+\frac{\sigma }{t} \int^{t}_{0}S(x)\,dB(x). $$

Let $M_{1}(t)=\int^{t}_{0}S(x)\,dB(x)$, which is a real-valued continuous local martingale, $M_{1}(0)=0$, and

$$ \limsup_{t\rightarrow\infty}\frac{\langle M_{1},M_{1}\rangle _{t}}{t}=\limsup_{t\rightarrow\infty} \frac{\int ^{t}_{0}S^{2}(x)\,dx}{t}\leq K^{2}< \infty \quad\mbox{a.s. } $$

Then by the strong law of large numbers we have

$$ \lim_{t\rightarrow\infty}\frac{M_{1}(t)}{t}=\lim_{t\rightarrow\infty } \frac{\int^{t}_{0}S(x)\,dB(x)}{t}=0 \quad\mbox{a.s.}, $$

which by (4.1) implies that

$$ \limsup_{t\rightarrow\infty}\frac{\log I(t)}{t}\leq\frac{\lambda ^{2}}{2\sigma^{2}}-\mu \quad\mbox{a.s. } $$

By condition (a) it is easy to see that

4.2 $$ \limsup_{t\rightarrow\infty}\frac{\log I(t)}{t}\leq \frac{\lambda^{2}}{2\sigma^{2}}-\mu< 0 \quad\mbox{a.s.}, $$

that is, $I(t)$ tends to zero exponentially almost surely. In other words, the infected prey population dies out with probability one.

(ii) $\frac{\lambda}{\sigma^{2}}\geq K$. Then we have

$$ f(S)\leq f(K)=\lambda K-\mu-\frac{\sigma^{2}K^{2}}{2}. $$

Therefore,

$$ d\log I\leq \biggl(\lambda K-\mu-\frac{\sigma^{2}K^{2}}{2} \biggr)\,dt+\sigma S\,dB(t). $$

Similarly, as in (i), we get

$$ \limsup_{t\rightarrow\infty}\frac{\log I(t)}{t}\leq\lambda K-\mu - \frac{\sigma^{2}K^{2}}{2} \quad\mbox{a.s. } $$

Using condition (b), we then obtain that

4.3 $$ \limsup_{t\rightarrow\infty}\frac{\log I(t)}{t}\leq\lambda K- \mu-\frac{\sigma^{2}K^{2}}{2}< 0 \quad\mbox{a.s.}, $$

that is, $I(t)$ tends to zero exponentially almost surely. In other words, the infected prey population dies out with probability one.

In the same way, by Itô’s formula we have

$$\begin{aligned} dP&=(-\theta\beta I P-\delta P+\theta\alpha S P)\,dt=P(-\theta\beta I -\delta+ \theta\alpha S)\,dt \\ &\leq P(-\theta\beta I -\delta+\theta\alpha K)\,dt\leq-P( \delta -\theta\alpha K)\,dt. \end{aligned}$$

Therefore,

$$ \limsup_{t\rightarrow\infty}\frac{\log P(t)}{t}\leq-( \delta-\theta \alpha K) \quad\mbox{a.s. } $$

Condition (c) implies

4.4 $$ \limsup_{t\rightarrow\infty}\frac{\log P(t)}{t}\leq-( \delta- \theta\alpha K)< 0 \quad\mbox{a.s.}, $$

thats is, $P(t)$ tends to zero exponentially almost surely. In other words, the predator population dies out with probability one.

By Itô’s formula we have

$$\begin{aligned} d\log S= \biggl[r-\frac{r}{K}S- \biggl(\frac{r}{K}+\lambda \biggr)I- \alpha P-\frac{\sigma ^{2}}{2}I^{2} \biggr]\,dt-\sigma I\,dB(t). \end{aligned}$$

Therefore,

$$\begin{aligned} \log S(t)-\log S(0)={}& rt-\frac{r}{K} \int^{t}_{0}S(s)\,ds- \biggl(\frac {r}{K}+ \lambda \biggr) \int^{t}_{0}I(s)\,ds- \alpha \int^{t}_{0}P(s)\,ds \\ &{}-\frac{\sigma^{2}}{2} \int^{t}_{0}I^{2}(s)\,ds-\sigma \int^{t}_{0}I(s)\,dB(s) \end{aligned}$$

and

$$\begin{aligned} \frac{r}{K}\frac{1}{t} \int^{t}_{0}S(s)\,ds ={}& r-\frac{\log S(t)-\log S(0)}{t} - \biggl(\frac{r}{K}+\lambda \biggr)\frac{1}{t} \int^{t}_{0}I(s)\,ds-\frac {\alpha}{t} \int^{t}_{0}P(s)\,ds \\ &{}-\frac{\sigma^{2}}{2}\frac{1}{t} \int^{t}_{0}I^{2}(s)\,ds-\sigma \frac {1}{t} \int^{t}_{0}I(s)\,dB(s) \\ \geq{}&r-\frac{\log K-\log S(0)}{t} - \biggl(\frac{r}{K}+\lambda \biggr) \frac{1}{t} \int ^{t}_{0}I(s)\,ds-\frac{\alpha}{t} \int^{t}_{0}P(s)\,ds \\ &{}-\frac{\sigma^{2}}{2}\frac{1}{t} \int^{t}_{0}I^{2}(s)\,ds-\sigma \frac {1}{t} \int^{t}_{0}I(s)\,dB(s). \end{aligned}$$

This, together with (4.2), (4.3), and (4.4), implies that

$$\begin{aligned} \liminf_{t\rightarrow\infty}\frac{1}{t} \int^{t}_{0}S(s)\,ds \geq K\quad \mbox{a.s. } \end{aligned}$$

Due to

$$\begin{aligned} K \leq\liminf_{t\rightarrow\infty}\frac{1}{t} \int^{t}_{0}S(s)\,ds \leq \limsup _{t\rightarrow\infty}\frac{1}{t} \int^{t}_{0}S(s)\,ds \leq K\quad \mbox{a.s.}, \end{aligned}$$

we obtain

$$\begin{aligned} \lim_{t\rightarrow\infty}\frac{1}{t} \int^{t}_{0}S(s)\,ds=K \quad\mbox{a.s. } \end{aligned}$$

 □

## Asymptotic behavior around the equilibrium $E_{2}=(\overline{S}, \overline{I}, 0)$ of system (1.1)

The equilibrium $E_{2}=(\overline{S}, \overline{I}, 0)$ of system (1.1) exists if $\lambda K>\mu$, but it is not an equilibrium of system (1.2). In this section, we first compute the convergence rate of $P(t)$. Then we study the fluctuations of system (1.2) about its equilibrium $E_{2}$ under some conditions.

### Theorem 5.1

*Let*$(S(t),I(t),P(t))$*be the solution of system* (1.2) *with initial value*$(S(0),I(0), P(0))\in\Gamma$. *If*$\lambda K >\mu$*and*$\alpha<\frac{\delta}{K \theta}$, *then*

5.1 $$ \begin{aligned} \limsup_{t\rightarrow\infty} \frac{\log P(t)}{t}\leq-(\delta-\theta \alpha K)< 0 \quad\textit{a.s.} \end{aligned} $$

*and*

$$ \limsup_{t\rightarrow\infty}\frac{1}{t} \int^{t}_{0} \bigl[ \bigl(S(s)-\overline {S} \bigr)^{2}+ \bigl(I(s)-\overline{I} \bigr)^{2} \bigr]\,ds\leq \frac{\sigma^{2}K^{2}(\overline {S}+\frac{\frac{r}{K}+\lambda}{\lambda}\overline{I}+\frac{\eta _{1}r^{2}}{\lambda^{2}})}{2m} \quad\textit{a.s.}, $$

*where*$E_{2}=(\overline{S}, \overline{I}, 0)$*is the boundary equilibrium of system* (1.1), $m=\min\{\frac{r}{2K},\frac{\mu\eta_{1}}{2}(\frac{\frac{r}{K}+\lambda }{\lambda})^{2}\}$, *and*$\eta_{1}=\frac{r}{2K}[r-\frac{r}{K}\overline{S}+\frac{r}{K}\frac{\frac{r}{K} +\lambda}{\lambda}\overline{I}+\frac{(r-\frac{2r}{K}\overline{S}-\mu )^{2}}{2\mu}]^{-1}$.

### Proof

By Itô’s formula, we can easily show that, for $t>0$,

$$\begin{aligned} dP&=(-\theta\beta I P-\delta P+\theta\alpha S P)\,dt=P(-\theta\beta I -\delta+ \theta\alpha S)\,dt \\ &\leq P(-\theta\beta I -\delta+\theta\alpha K)\,dt\leq-P( \delta -\theta\alpha K)\,dt. \end{aligned}$$

Therefore,

$$ \limsup_{t\rightarrow\infty}\frac{\log P(t)}{t}\leq-( \delta-\theta \alpha K) \quad \mbox{a.s. } $$

It then follows from the condition $\alpha<\frac{\delta}{K \theta}$ that

$$ \limsup_{t\rightarrow\infty}\frac{\log P(t)}{t}\leq-( \delta-\theta\alpha K)< 0 \quad \mbox{a.s.}, $$

that is, $P(t)$ tends to zero exponentially almost surely. In other words, the predator population dies out with probability one. That is to say, we can see that $\lim_{t\rightarrow\infty} P(t)=0$.

Since $(\overline{S}, \overline{I}, 0)$ is the boundary equilibrium of system (1.1), we have

$$r \biggl(1-\frac{\overline{S}+\overline{I}}{K} \biggr)=\lambda \overline{I},\qquad \mu= \lambda \overline{S}. $$

Define

$$\begin{aligned} V(S,I,P)&=S-\overline{S}-\overline{S} \log\frac{S}{\overline{S}}+\frac {\frac{r}{K}+\lambda}{\lambda} \biggl(I-\overline{I}-\overline{I} \log\frac {I}{\overline{I}} \biggr)+ \frac{\eta_{1}}{2} \biggl[S-\overline{S}+\frac{\frac {r}{K}+\lambda}{\lambda}(I-\overline{I}) \biggr]^{2} \\ &:=V_{1}+\eta_{1}V_{2}, \end{aligned}$$

where $\eta_{1}$ is a positive constant, which is determined later. By Itô’s formula and (1.2) we compute

$$\begin{aligned} dV_{1}={}& \biggl\{ (S-\overline{S}) \biggl[r \biggl(1- \frac{S+I}{K} \biggr)-\lambda I-\alpha P \biggr]+\frac {\sigma^{2}I^{2}}{2} \overline{S} \biggr\} \,dt- \sigma(S-\overline{S})I\,dB(t) \\ &{}+\frac{\frac{r}{K}+\lambda}{\lambda} \biggl\{ \biggl[(I-\overline{I}) (\lambda S-\beta P-\mu)+ \frac{\sigma^{2}S^{2}}{2}\overline{I} \biggr]\,dt+\sigma (I-\overline{I})S\,dB(t) \biggr\} \\ :={}&LV_{1}\,dt+ \biggl[\frac{\frac{r}{K}+\lambda}{\lambda}\sigma(I-\overline {I})S- \sigma(S-\overline{S})I \biggr]\,dB(t), \end{aligned}$$

where

$$\begin{aligned} LV_{1}={}&(S-\overline{S}) \biggl[r \biggl(1-\frac{S+I}{K} \biggr)- \lambda I-\alpha P \biggr]+\frac {\sigma^{2}I^{2}}{2}\overline{S} \\ &{}+\frac{\frac{r}{K}+\lambda}{\lambda } \biggl[(I-\overline{I}) (\lambda S-\beta P-\mu)+ \frac{\sigma ^{2}S^{2}}{2}\overline{I} \biggr] \\ ={}&-\frac{r}{K}(S-\overline{S})^{2}- \biggl( \frac{r}{K}+ \lambda \biggr) (S-\overline {S}) (I-\overline{I})-\alpha P(S- \overline{S})+ \frac{\sigma ^{2}I^{2}}{2}\overline{S} \\ &{}+\frac{\frac{r}{K}+\lambda}{\lambda} \biggl[\lambda (S-\overline{S}) (I-\overline{I}) -\beta P(I-\overline{I})+\frac{\sigma^{2}S^{2}}{2}\overline{I} \biggr] \\ ={}&-\frac{r}{K}(S-\overline{S})^{2}-\alpha P(S-\overline{S})- \frac{\frac {r}{K}+\lambda}{\lambda}\beta P(I-\overline{I})+\frac{\sigma ^{2}I^{2}}{2}\overline{S}+ \frac{\frac{r}{K}+\lambda}{\lambda}\frac {\sigma^{2}S^{2}}{2}\overline{I}. \end{aligned}$$

Let $Y=S-\overline{S}+\frac{\frac{r}{K}+\lambda}{\lambda}(I-\overline {I})$. Then

$$\begin{aligned}& dY= \biggl(rS-\frac{r}{K}S^{2}-\alpha SP-\frac{\frac{r}{K}+\lambda}{\lambda } \beta IP-\frac{\frac{r}{K}+\lambda}{\lambda}\mu I \biggr)\,dt+\frac{r\sigma }{K\lambda}SI\,dB(t),\\& \begin{aligned}[b] dV_{2}={}& \biggl[S-\overline{S}+\frac{\frac{r}{K}+\lambda}{\lambda}(I-\overline {I}) \biggr] \biggl[ \biggl(rS-\frac{r}{K}S^{2}-\alpha SP- \frac{\frac{r}{K}+\lambda}{\lambda }\beta IP-\frac{\frac{r}{K}+\lambda}{\lambda}\mu I \biggr)\,dt \\ &{}+\frac{r\sigma }{K\lambda}SI\,dB(t) \biggr]+\frac{r^{2}\sigma^{2}}{2K^{2}\lambda ^{2}}S^{2}I^{2}\,dt \\ :={}&LV_{2}\,dt+ \biggl[S-\overline{S}+\frac{\frac{r}{K}+\lambda}{\lambda }(I- \overline{I}) \biggr]\frac{r\sigma}{K\lambda}SI\,dB(t), \end{aligned} \end{aligned}$$

where

$$\begin{aligned} LV_{2}={}& \biggl[S-\overline{S}+\frac{\frac{r}{K}+\lambda}{\lambda}(I-\overline {I}) \biggr] \biggl(rS-\frac{r}{K}S^{2}-\alpha SP-\frac{\frac{r}{K}+\lambda}{\lambda } \beta IP-\frac{\frac{r}{K}+\lambda}{\lambda}\mu I \biggr) \\ &{}+\frac{r^{2}\sigma ^{2}}{2K^{2}\lambda^{2}}S^{2}I^{2} \\ ={}& \biggl[S-\overline{S}+\frac{\frac{r}{K}+\lambda}{\lambda}(I-\overline {I}) \biggr] \biggl[- \frac{r}{K}(S-\overline{S})^{2} + \biggl(r-\frac{2r}{K} \overline{S} \biggr) (S-\overline{S})-\alpha SP \\ &{}-\frac{\frac {r}{K}+\lambda}{\lambda}\beta P(I-\overline{I}) -\frac{\frac{r}{K}+\lambda}{\lambda}\mu(I- \overline{I})-\frac{\frac {r}{K}+\lambda}{\lambda}\beta\overline{I}P \biggr]+\frac{r^{2}\sigma ^{2}}{2K^{2}\lambda^{2}}S^{2}I^{2} \\ ={}&-\frac{r}{K}S(S-\overline{S})^{2}+ \biggl(r- \frac{r}{K}\overline{S}+\frac {r}{K}\frac{\frac{r}{K} +\lambda}{\lambda}\overline{I} \biggr) (S-\overline{S})^{2}-\frac{\frac {r}{K}+\lambda}{\lambda}\beta P(S-\overline{S}) (I-\overline{I}) \\ &{}-\alpha SP(S-\overline{S})-\frac{\frac{r}{K}+\lambda}{\lambda}\beta \overline{I} P(S- \overline{S})+\frac{\frac{r}{K}+\lambda}{\lambda } \biggl(r-\frac{2r}{K}\overline{S}-\mu \biggr) (S-\overline{S}) (I-\overline{I}) \\ &{}-\frac{r}{K}\frac{\frac{r}{K}+\lambda}{\lambda}I(S-\overline {S})^{2}- \frac{\frac{r}{K}+\lambda}{\lambda}\alpha SP(I-\overline {I})- \biggl(\frac{\frac{r}{K}+\lambda}{\lambda} \biggr)^{2}\beta P(I-\overline {I})^{2} \\ &{}- \biggl(\frac{\frac{r}{K}+\lambda}{\lambda} \biggr)^{2}\beta\overline {I}P(I- \overline{I}) -\mu \biggl(\frac{\frac{r}{K}+\lambda}{\lambda} \biggr)^{2}(I- \overline{I})^{2}+\frac {r^{2}\sigma^{2}}{2K^{2}\lambda^{2}}S^{2}I^{2} \\ \leq{}& \biggl(r-\frac{r}{K}\overline{S}+\frac{r}{K} \frac{\frac{r}{K} +\lambda}{\lambda}\overline{I} \biggr) (S-\overline{S})^{2}+ \frac{\frac {r}{K}+\lambda}{\lambda} \biggl(r-\frac{2r}{K}\overline{S}-\mu \biggr) (S- \overline {S}) (I-\overline{I}) \\ &{}-\mu \biggl(\frac{\frac{r}{K}+\lambda}{\lambda} \biggr)^{2}(I-\overline{I})^{2} -\frac{\frac{r}{K}+\lambda}{\lambda}\beta P(S-\overline{S}) (I-\overline {I})-\alpha SP(S- \overline{S}) \\ &{}-\frac{\frac{r}{K}+\lambda}{\lambda }\beta\overline{I} P(S-\overline{S})-\frac{\frac{r}{K}+\lambda}{\lambda } \alpha S P(I-\overline{I}) - \biggl(\frac{\frac{r}{K}+\lambda}{\lambda} \biggr)^{2}\beta\overline {I}P(I- \overline{I}) +\frac{r^{2}\sigma^{2}}{2K^{2}\lambda^{2}}S^{2}I^{2}. \end{aligned}$$

By the Cauchy inequality we can easily show that

$$\begin{aligned} LV_{2}\leq{}& \biggl[r-\frac{r}{K}\overline{S}+ \frac{r}{K}\frac{\frac {r}{K}+\lambda}{\lambda}\overline{I}+\frac{(r-\frac{2r}{K}\overline {S}-\mu)^{2}}{2\mu} \biggr](S- \overline{S})^{2} -\frac{\mu}{2} \biggl(\frac{\frac{r}{K}+\lambda}{\lambda} \biggr)^{2}(I-\overline {I})^{2} \\ &{}-\frac{\frac{r}{K}+\lambda}{\lambda}\beta P(S-\overline{S}) (I-\overline {I})-\alpha SP(S- \overline{S})-\frac{\frac{r}{K}+\lambda}{\lambda}\beta \overline{I} P(S-\overline{S}) \\ &{}-\frac{\frac{r}{K}+\lambda}{\lambda }\alpha SP(I-\overline{I})- \biggl(\frac{\frac{r}{K}+\lambda}{\lambda } \biggr)^{2}\beta\overline{I}P(I-\overline{I}) +\frac{r^{2}\sigma^{2}}{2K^{2}\lambda^{2}}S^{2}I^{2}. \end{aligned}$$

By choosing $\eta_{1}$ such that $\eta_{1}[r-\frac{r}{K}\overline {S}+\frac{r}{K}\frac{\frac{r}{K}+\lambda}{\lambda}\overline{I}+\frac {(r-\frac{2r}{K}\overline{S}-\mu)^{2}}{2\mu}]=\frac{r}{2K}$, that is, $\eta_{1}=\frac{r}{2K}[r-\frac{r}{K}\overline{S}+\frac{r}{K}\frac{\frac {r}{K}+\lambda}{\lambda}\overline{I} +\frac{(r-\frac{2r}{K}\overline{S}-\mu)^{2}}{2\mu}]^{-1}$, we see that

$$\begin{aligned} LV={}&LV_{1}+\eta_{1}LV_{2} \\ \leq{}& {-}\frac{r}{2K}(S-\overline{S})^{2}-\frac{\mu\eta_{1}}{2} \biggl(\frac{\frac {r}{K}+\lambda}{\lambda} \biggr)^{2}(I-\overline{I})^{2}- \alpha P(S-\overline {S})-\frac{\frac{r}{K}+\lambda}{\lambda}\beta P(I-\overline{I}) \\ &{}+\eta_{1} \biggl[-\frac{\frac{r}{K}+\lambda}{\lambda}\beta P(S-\overline {S}) (I- \overline{I})-\alpha SP(S-\overline{S})-\frac{\frac{r}{K}+\lambda }{\lambda}\beta\overline{I} P(S- \overline{S}) \\ &{}-\frac{\frac{r}{K}+\lambda}{\lambda}\alpha S P(I-\overline{I})- \biggl(\frac {\frac{r}{K}+\lambda}{\lambda} \biggr)^{2}\beta\overline{I}P(I-\overline{I}) \biggr] + \frac{\sigma^{2}I^{2}}{2}\overline{S}+\frac{\frac{r}{K}+\lambda }{\lambda}\frac{\sigma^{2}S^{2}}{2}\overline{I} \\ &{}+\frac{\eta_{1}r^{2}\sigma^{2}}{2K^{2}\lambda^{2}}S^{2}I^{2}. \end{aligned}$$

Notice that

$$\begin{aligned} dV={}&LV\,dt+\frac{\frac{r}{K}+\lambda}{\lambda}\sigma(I-\overline {I})S\,dB(t)-\sigma(S- \overline{S})I\,dB(t) \\ &{}+\frac{\eta_{1}r}{K\lambda}\sigma SI \biggl[S-\overline{S}+\frac{\frac {r}{K}+\lambda}{\lambda}(I- \overline{I}) \biggr]\,dB(t) \\ ={}&LV\,dt+ \biggl[\frac{\frac{r}{K}+\lambda}{\lambda}\sigma S(I-\overline {I})-\sigma I(S- \overline{S})+\frac{\eta_{1}r}{K\lambda}\sigma SI(S-\overline{S}) \\ &{}+\frac{\frac{r}{K}+\lambda}{\lambda}\frac{\eta_{1}r}{K\lambda}\sigma SI(I-\overline{I}) \biggr]\,dB(t). \end{aligned}$$

Integrating both sides of from 0 to *t* yields

5.2 $$\begin{aligned} &V(t)-V(0) \\ &\quad\leq-\frac{r}{2K} \int^{t}_{0} \bigl(S(s)-\overline{S} \bigr)^{2}\,ds-\frac{\mu\eta _{1}}{2} \biggl(\frac{\frac{r}{K}+\lambda}{\lambda} \biggr)^{2} \int ^{t}_{0} \bigl(I(s)-\overline{I} \bigr)^{2}\,ds \\ &\qquad{}- \int^{t}_{0}\alpha P(s) \bigl(S(s)-\overline{S} \bigr)\,ds \\ &\qquad{}- \int^{t}_{0}\frac{\frac{r}{K}+\lambda}{\lambda}\beta P(s) \bigl(I(s)-\overline{I} \bigr)\,ds+\eta_{1} \biggl[- \int^{t}_{0}\frac{\frac {r}{K}+\lambda}{\lambda}\beta P(s) \bigl(S(s)-\overline{S} \bigr) \bigl(I(s)-\overline {I} \bigr)\,ds \\ &\qquad{}- \int^{t}_{0}\alpha S(s)P(s) \bigl(S(s)-\overline{S} \bigr)\,ds- \int^{t}_{0}\frac {\frac{r}{K}+\lambda}{\lambda}\beta\overline{I} P(s) \bigl(S(s)-\overline {S} \bigr)\,ds \\ &\qquad{}- \int^{t}_{0}\frac{\frac{r}{K}+\lambda}{\lambda}\alpha S(s)P(s) \bigl(I(s)-\overline{I} \bigr)\,ds- \int^{t}_{0} \biggl(\frac{\frac{r}{K}+\lambda }{\lambda} \biggr)^{2}\beta\overline{I}P(s) \bigl(I(s)-\overline{I} \bigr)\,ds \biggr] \\ &\qquad{}+\frac{\sigma^{2}K^{2}}{2} \biggl(\overline{S}+\frac{\frac{r}{K}+\lambda }{\lambda}\overline{I}+ \frac{\eta_{1}r^{2}}{\lambda^{2}} \biggr)t + \int^{t}_{0}\sigma \biggl[\frac{\frac{r}{K}+\lambda}{\lambda }S(s) \bigl(I(s)-\overline{I} \bigr)-I(s) \bigl(S(s)-\overline{S} \bigr) \\ &\qquad{}+\frac{\eta_{1}r}{K\lambda} S(s)I(s) \bigl(S(s)-\overline{S} \bigr)+ \frac{\frac {r}{K}+\lambda}{\lambda}\frac{\eta_{1}r}{K\lambda }S(s)I(s) \bigl(I(s)-\overline{I} \bigr) \biggr]\,dB(s). \end{aligned}$$

Let

$$\begin{aligned} f(t)={}&- \int^{t}_{0}\alpha P(s) \bigl(S(s)-\overline{S} \bigr)\,ds- \int^{t}_{0}\frac {\frac{r}{K}+\lambda}{\lambda}\beta P(s) \bigl(I(s)-\overline{I} \bigr)\,ds \\ &{}+\eta_{1} \biggl[- \int^{t}_{0}\frac{\frac{r}{K}+\lambda}{\lambda}\beta P(s) \bigl(S(s)-\overline{S} \bigr) \bigl(I(s)-\overline{I} \bigr)\,ds- \int^{t}_{0}\alpha S(s)P(s) \bigl(S(s)-\overline{S} \bigr)\,ds \\ &{}- \int^{t}_{0}\frac{\frac{r}{K}+\lambda}{\lambda}\beta\overline{I} P(s) \bigl(S(s)-\overline{S} \bigr)\,ds- \int^{t}_{0}\frac{\frac{r}{K}+\lambda}{\lambda }\alpha S(s)P(s) \bigl(I(s)-\overline{I} \bigr)\,ds \\ &{}- \int^{t}_{0} \biggl(\frac{\frac{r}{K}+\lambda}{\lambda} \biggr)^{2}\beta\overline {I}P(s) \bigl(I(s)-\overline{I} \bigr)\,ds \biggr]. \end{aligned}$$

By the boundedness of $S(t)$ and $I(t)$ and by (5.1) we can show that

$$\begin{aligned} \limsup_{t\rightarrow\infty}\frac{f(t)}{t}=0 \quad\mbox{a.s.} \end{aligned}$$

Let

$$\begin{aligned} M_{2}(t)={}& \int^{t}_{0}\sigma \biggl[\frac{\frac{r}{K}+\lambda}{\lambda }S(s) \bigl(I(s)-\overline{I}(s) \bigr)-I(s) \bigl(S(s)-\overline{S}(s) \bigr)+ \frac{\eta _{1}r}{K\lambda} S(s)I(s) \bigl(S(s)-\overline{S}(s) \bigr) \\ &{}+\frac{\frac{r}{K}+\lambda}{\lambda}\frac{\eta_{1}r}{K\lambda }S(s)I(s) \bigl(I(s)-\overline{I}(s) \bigr) \biggr]\,dB(s), \end{aligned}$$

which is a real-valued continuous local martingale, $M_{2}(0)=0$, and

$$\begin{aligned} &\limsup_{t\rightarrow\infty}\frac{\langle M_{2},M_{2} \rangle_{t}}{t} \\ &\quad=\limsup_{t\rightarrow\infty}\frac{1}{t} \int^{t}_{0}\sigma^{2} \biggl[ \frac {\frac{r}{K}+\lambda}{\lambda}S(s) \bigl(I(s)-\overline {I} \bigr)-I(s) \bigl(S(s)- \overline{S} \bigr) +\frac{r\eta_{1}}{K\lambda}S(s)I(s) \bigl(S(s)-\overline{S} \bigr) \\ &\qquad{}+\frac{r\eta _{1}}{K\lambda}\frac{\frac{r}{K}+\lambda}{\lambda }S(s)I(s) \bigl(I(s)-\overline{I} \bigr) \biggr]^{2}\,ds \\ \leq&16K^{4}\sigma^{2} \biggl[ \biggl(\frac{\frac{r}{K} +\lambda}{\lambda} \biggr)^{2}+1+\frac{r^{2}\eta_{1}^{2}}{\lambda^{2}} \biggl(1+ \biggl(\frac {\frac{r}{K}+\lambda}{\lambda} \biggr)^{2} \biggr) \biggr]< \infty \quad\mbox{a.s.} \end{aligned}$$

Then by the strong law of large numbers we have

$$ \lim_{t\rightarrow\infty}\frac{M_{2}(t)}{t}=0 \quad\mbox{a.s.} $$

It then follows from (5.2) that

$$\begin{aligned} &\limsup_{t\rightarrow\infty}\frac{1}{t} \int^{t}_{0} \biggl[\frac {r}{2K} \bigl(S(s)- \overline{S} \bigr)^{2}+\frac{\mu\eta_{1}}{2} \biggl(\frac{\frac {r}{K}+\lambda}{\lambda} \biggr)^{2} \bigl(I(s)-\overline{I} \bigr)^{2} \biggr]\,ds \\ &\quad\leq \frac{\sigma^{2}K^{2}(\overline{S}+\frac{\frac{r}{K}+\lambda }{\lambda}\overline{I}+\frac{\eta_{1}r^{2}}{\lambda^{2}})}{2} \quad\mbox{a.s.} \end{aligned}$$

Then we obtain

$$\begin{aligned} &\limsup_{t\rightarrow\infty}\frac{1}{t} \int^{t}_{0} \bigl[ \bigl(S(s)-\overline {S} \bigr)^{2}+ \bigl(I(s)-\overline{I} \bigr)^{2} \bigr]\,ds\\ &\quad\leq \frac{\sigma^{2}K^{2}(\overline {S}+\frac{\frac{r}{K}+\lambda}{\lambda}\overline{I}+\frac{\eta _{1}r^{2}}{\lambda^{2}})}{2m} \quad\mbox{a.s.}, \end{aligned}$$

where $m=\min\{\frac{r}{2K},\frac{\mu\eta_{1}}{2}(\frac{\frac {r}{K}+\lambda}{\lambda})^{2}\}$.

Hence, the proof of this theorem is completed. □

## Stochastic asymptotic stability around the equilibrium $E_{3}=(\widetilde{S},0,\widetilde{P})$

Since $(\widetilde{S},0,\widetilde{P})$ is the boundary equilibrium of system (1.1), we have

$$r \biggl(1-\frac{\widetilde{S}}{K} \biggr)=\alpha\widetilde{P},\qquad \delta=\theta \alpha\widetilde{S}. $$

The stochastic system (1.2) can be centered at its equilibrium $E_{3}=(\widetilde{S},0,\widetilde{P})$ by the change of variables

$$u=S-\widetilde{S},\qquad w=P-\widetilde{P}. $$

We obtain the following system:

6.1 $$ \left \{ \textstyle\begin{array}{@{}l} du= [(r-\frac{2r}{K}\widetilde{S}-\alpha\widetilde{P})u-(\frac {r}{K}+\lambda)\widetilde{S}I-\alpha\widetilde{S}w-\frac{r}{K}u^{2} -(\frac{r}{K}+\lambda)uI-\alpha uw]\,dt\\ \hphantom{du=}{}-(\sigma uI+\sigma\widetilde {S}I)\,dB(t),\\ dI= [(\lambda\widetilde{S}-\beta\widetilde{P}-\mu)I+\lambda u I-\beta Iw]\,dt+(\sigma uI+\sigma\widetilde{S}I)\,dB(t),\\ dw=(\theta\alpha\widetilde{P}u-\theta\beta\widetilde{P}I-\theta\beta Iw+\theta\alpha uw)\,dt. \end{array}\displaystyle \right . $$

It is easy to see that the stability of the equilibrium of system (1.2) is equivalent to the stability of the zero solution of system (6.1).

### Theorem 6.1

*Let*$(S(t),I(t),P(t))$*be the solution of system* (1.2) *with initial value*$(S(0),I(0), P(0))\in\Gamma$. *If*

$$ \alpha>\frac{\delta\lambda}{\mu\theta},\qquad \lambda>\frac{\mu}{K},\qquad \sigma ^{2}< \frac{2(\beta\widetilde{P}+\mu-\lambda\widetilde{S})}{\widetilde{S}^{2}}, $$

*then*$E_{3}=(\widetilde{S}, 0, \widetilde{P})$*is stochastically asymptotically stable*.

### Proof

It is easy to see that we only need to prove that the zero solution of (6.1) is stochastically asymptotically stable.

Let $x=(u,I,w)$. Define the Lyapunov function $V(x)$ as follows:

$$\begin{aligned} V(x)&=\eta_{2}\frac{1}{2} \biggl(u+I+\frac{1}{\theta}w \biggr)^{2}+\frac {1}{2}(u+I)^{2}+\eta_{1} \frac{1}{2}w^{2}+\eta_{3}\frac{1}{2}I^{2} \\ &:=\eta_{2}V_{1}+V_{2}+\eta_{1}V_{4}+ \eta_{3}V_{3}, \end{aligned}$$

where $\eta_{1}$, $\eta_{2}$, $\eta_{3}$ are positive constants, which are determined later. By Itô’s formula we compute

$$\begin{aligned} dV_{1}={}& \biggl(u+I+\frac{1}{\theta}w \biggr) \biggl[ \biggl(r- \frac{2r}{K}\widetilde{S} \biggr)u- \biggl(\frac {r}{K} \widetilde{S}+2 \beta\widetilde{P}+\mu \biggr)I\\ &{}-\alpha\widetilde{S}w- \frac {r}{K}u^{2} -\frac{r}{K}uI-2\beta Iw \biggr]\,dt, \end{aligned}$$

where

$$\begin{aligned} LV_{1}={}& \biggl(u+I+\frac{1}{\theta}w \biggr) \biggl[ \biggl(r- \frac{2r}{K}\widetilde{S} \biggr)u- \biggl(\frac {r}{K} \widetilde{S}+2 \beta\widetilde{P}+\mu \biggr)I-\alpha\widetilde{S}w- \frac {r}{K}u^{2} -\frac{r}{K}uI-2\beta Iw \biggr] \\ ={}& \biggl(r-\frac{2r}{K}\widetilde{S} \biggr)u^{2}- \biggl( \frac{r}{K}\widetilde{S}+2\beta \widetilde{P}+\mu \biggr)I^{2}- \frac{\delta}{\theta^{2}}w^{2} + \biggl(r-\frac{3r}{K}\widetilde{S}-2 \beta\widetilde{P}-\mu \biggr)uI \\ &{}+\frac {1}{\theta} \biggl(r-\frac{2r}{K}\widetilde{S} -\delta \biggr)uw-\frac{1}{\theta} \biggl(\frac{r}{K}\widetilde{S}+2\beta \widetilde {P}+\mu+\delta \biggr)Iw \\ &{}+ \biggl(u+I+\frac{1}{\theta}w \biggr) \biggl(-\frac{r}{K}u^{2}- \frac{r}{K}uI-2\beta Iw \biggr). \end{aligned}$$

Since $(\widetilde{S},0,\widetilde{P})$ is the boundary equilibrium of system (1.1), we get

$$r-\frac{2r}{K}\widetilde{S}=\alpha\widetilde{P}-\frac{r}{K} \widetilde {S}\leq\alpha\widetilde{P}. $$

Moreover, using the Cauchy inequality, we obtain

$$\begin{aligned} LV_{1}\leq{}& \alpha\widetilde{P}u^{2}- \biggl( \frac{r}{K}\widetilde{S}+2\beta\widetilde {P}+\mu \biggr)I^{2}- \frac{\delta}{\theta^{2}}w^{2} + \biggl(r-\frac{3r}{K}\widetilde{S}-2 \beta\widetilde{P}-\mu \biggr)uI \\ &{}+\frac{1}{\theta} \biggl(r-\frac{2r}{K}\widetilde{S} -\delta \biggr)uw-\frac{1}{\theta} \biggl(\frac{r}{K}\widetilde{S}+2\beta \widetilde {P}+\mu+\delta \biggr)Iw \\ &{}+ \biggl(u+I+\frac{1}{\theta}w \biggr) \biggl(-\frac{r}{K}u^{2}- \frac{r}{K}uI-2\beta Iw \biggr) \\ \leq{}& \biggl[\alpha\widetilde{P}+\frac{1}{\delta} \biggl(r- \frac{2r}{K}\widetilde {S}-\delta \biggr)^{2} + \frac{1}{4} \biggl(r-\frac{3r}{K}\widetilde{S}-2\beta\widetilde{P}- \mu \biggr)^{2} \biggl(\frac{r}{K}\widetilde{S}+2\beta \widetilde{P}+ \mu \biggr)^{-1} \biggr]u^{2} \\ &+\frac{1}{\delta} \biggl(\frac{r}{K}\widetilde{S}+2\beta \widetilde{P}+ \mu +\delta \biggr)^{2}I^{2}- \frac{\delta}{2\theta^{2}}w^{2} \\ &{}+ \biggl(u+I+\frac {1}{\theta}w \biggr) \biggl(-\frac{r}{K}u^{2}- \frac{r}{K}uI-2\beta Iw \biggr). \end{aligned}$$

Further,

$$\begin{aligned} dV_{2}={}&(u+I) \biggl[ \biggl(r-\frac{2r}{K}\widetilde{S}- \alpha \widetilde{P} \biggr)u- \biggl(\frac {r}{K}\widetilde{S}+\beta \widetilde{P}+ \mu \biggr)I-\alpha\widetilde{S}w-\frac {r}{K}u^{2} \\ &{}-\frac{r}{K}uI-\alpha uw-\beta Iw \biggr]\,dt, \end{aligned}$$

where

$$\begin{aligned}& \begin{aligned}[b] LV_{2}={}&(u+I) \biggl[ \biggl(r- \frac{2r}{K} \widetilde{S}-\alpha\widetilde{P} \biggr)u- \biggl( \frac {r}{K}\widetilde{S}+ \beta\widetilde{P}+\mu \biggr)I-\alpha \widetilde{S}w-\frac {r}{K}u^{2} -\frac{r}{K}uI \\ &{}-\alpha uw-\beta Iw \biggr] \\ ={}& \biggl(r-\frac{2r}{K}\widetilde{S}-\alpha\widetilde{P} \biggr)u^{2}- \biggl(\frac {r}{K}\widetilde{S}+\beta\widetilde{P}+ \mu \biggr)I^{2}+ \biggl(r-\frac{3r}{K}\widetilde{S}-\alpha \widetilde{P}-\beta\widetilde{P}-\mu \biggr)uI \\ &{}-\frac{\delta}{\theta}uw-\frac{\delta}{\theta}Iw +(u+I) \biggl(- \frac{r}{K}u^{2}-\frac{r}{K}uI-\alpha uw-\beta Iw \biggr) \\ ={}&{-}\frac{r}{K}\widetilde{S}u^{2}- \biggl(\frac{r}{K} \widetilde{S}+\beta \widetilde{P}+\mu \biggr)I^{2}+ \biggl(r- \frac{3r}{K}\widetilde{S}-\alpha\widetilde{P}-\beta\widetilde{P}-\mu \biggr)uI \\ &{}-\frac{\delta}{\theta}uw-\frac{\delta}{\theta}Iw +(u+I) \biggl(- \frac{r}{K}u^{2}-\frac{r}{K}uI-\alpha uw-\beta Iw \biggr); \end{aligned} \\& \begin{aligned}[b] dV_{3}={}&I \bigl\{ \bigl[(\lambda\widetilde{S}- \beta \widetilde{P}-\mu)I+\lambda u I-\beta Iw \bigr]\,dt+(\sigma uI+\sigma \widetilde{S}I)\,dB(t) \bigr\} \\ &{}+\frac{1}{2}(\sigma uI+\sigma\widetilde{S}I)^{2}\,dt, \end{aligned} \end{aligned}$$

where

$$\begin{aligned} LV_{3}&=I \bigl[(\lambda\widetilde{S}-\beta\widetilde{P}-\mu)I+\lambda u I-\beta Iw \bigr]+\frac{1}{2}(\sigma uI+\sigma\widetilde{S}I)^{2} \\ &= \biggl(\lambda\widetilde{S}-\beta\widetilde{P}-\mu+\frac{1}{2}\sigma ^{2}\widetilde{S}^{2} \biggr)I^{2}+ \biggl(\lambda u- \beta w+\frac{1}{2}\sigma ^{2}u^{2}+ \sigma^{2}\widetilde{S}u \biggr)I^{2}. \end{aligned}$$

By the condition $\sigma^{2}<\frac{2(\beta\widetilde{P}+\mu-\lambda \widetilde{S})}{\widetilde{S}^{2}}$ we get that $\lambda\widetilde {S}-\beta\widetilde{P}-\mu+\frac{1}{2}\sigma^{2}\widetilde{S}^{2}<0$.

We further have

$$\begin{aligned} dV_{4}=w(\theta\alpha\widetilde{P}u-\theta\beta\widetilde{P}I-\theta \beta Iw+\theta\alpha uw)\,dt, \end{aligned}$$

where

$$\begin{aligned} LV_{4}&=w(\theta\alpha\widetilde{P}u-\theta\beta\widetilde{P}I-\theta \beta Iw+\theta\alpha uw) =\theta\alpha\widetilde{P}uw-\theta\beta\widetilde{P}Iw+w(-\theta \beta Iw+ \theta\alpha uw). \end{aligned}$$

Then we obtain

6.2 $$\begin{aligned} &LV_{2}+\eta_{1}LV_{4} \\ &\quad=-\frac{r}{K}\widetilde{S}u^{2}- \biggl( \frac{r}{K} \widetilde{S}+\beta \widetilde{P}+\mu \biggr)I^{2}+ \biggl(r- \frac{3r}{K}\widetilde{S}-\alpha\widetilde{P}-\beta\widetilde{P}- \mu \biggr)uI- \biggl(\frac{\delta}{\theta}+\eta_{1}\theta\beta \widetilde{P} \biggr)Iw \\ &\qquad{}+ \biggl(\eta_{1}\theta\alpha\widetilde{P}- \frac{\delta}{\theta} \biggr)uw+M_{1}(u,I,w), \end{aligned}$$

where

$$\begin{aligned} M_{1}(u,I,w)=(u+I) \biggl(-\frac{r}{K}u^{2}- \frac{r}{K}uI-\alpha uw-\beta Iw \biggr)+\eta_{1}w(-\theta\beta Iw+\theta\alpha uw). \end{aligned}$$

In (6.2), we choose $\eta_{1}=\frac{\delta}{\alpha\widetilde {P}\theta^{2}}$ such that $\eta_{1}\theta\alpha\widetilde{P}-\frac {\delta}{\theta}=0$.

Moreover, using the Cauchy inequality, we obtain

6.3 $$ \biggl(r-\frac{3r}{K}\widetilde{S}-\alpha\widetilde{P}- \beta\widetilde{P}-\mu \biggr)uI \leq\frac{r\widetilde{S}}{2K}u^{2}+ \frac{K}{2r\widetilde{S}} \biggl(r-\frac {3r}{K}\widetilde{S}-\alpha\widetilde{P}- \beta\widetilde{P}-\mu \biggr)^{2}I^{2}. $$

Substituting (6.3) into (6.2) yields

$$\begin{aligned} &LV_{2}+\eta_{1}LV_{4} \\ &\quad\leq-\frac{r\widetilde{S}}{2K}u^{2}+ \frac{K}{2r\widetilde{S}} \biggl(r- \frac{3r}{K}\widetilde{S}-\alpha\widetilde {P}-\beta\widetilde{P}-\mu \biggr)^{2}I^{2}- \biggl(\frac{r}{K}\widetilde{S}+\beta \widetilde{P}+\mu \biggr)I^{2}\\ &\qquad{}- \biggl(\frac{\delta}{\theta}+ \eta_{1}\theta\beta \widetilde{P} \biggr)Iw +M_{1}(u,I,w), \end{aligned}$$

and so we have

$$\begin{aligned} &\eta_{2}LV_{1}+LV_{2}+\eta_{1}LV_{4} \\ &\quad\leq \eta_{2} \biggl\{ \biggl[\alpha\widetilde{P}+ \frac{1}{\delta} \biggl(r-\frac {2r}{K}\widetilde{S}-\delta \biggr)^{2} +\frac{1}{4} \biggl(r-\frac{3r}{K} \widetilde{S}-2\beta\widetilde{P}-\mu \biggr)^{2} \biggl( \frac{r}{K}\widetilde{S}+2\beta\widetilde{P}+\mu \biggr)^{-1} \biggr]u^{2} \\ &\qquad{}+\frac{1}{\delta} \biggl(\frac{r}{K}\widetilde{S}+2\beta \widetilde{P}+\mu +\delta \biggr)^{2}I^{2}- \frac{\delta}{2\theta^{2}}w^{2}+ \biggl(u+I+\frac{1}{\theta }w \biggr) \biggl(- \frac{r}{K}u^{2}-\frac{r}{K}uI-2\beta Iw \biggr) \biggr\} \\ &\qquad{}-\frac{r\widetilde{S}}{2K}u^{2} +\frac{K}{2r\widetilde{S}} \biggl(r- \frac{3r}{K}\widetilde{S}-\alpha\widetilde {P}-\beta\widetilde{P}-\mu \biggr)^{2}I^{2} - \biggl(\frac{r}{K}\widetilde{S}+ \beta \widetilde{P}+\mu \biggr)I^{2} \\ &\qquad{}- \biggl(\frac{\delta}{\theta}+\eta_{1}\theta\beta\widetilde{P} \biggr)Iw +M_{1}(u,I,w) \\ &\quad\leq \biggl\{ \eta_{2} \biggl[\alpha\widetilde{P}+\frac{1}{\delta} \biggl(r-\frac {2r}{K}\widetilde{S}-\delta \biggr)^{2} \\ &\qquad{}+ \frac{1}{4} \biggl(r-\frac{3r}{K}\widetilde{S}-2\beta\widetilde{P}- \mu \biggr)^{2} \biggl(\frac{r}{K}\widetilde{S}+2\beta \widetilde{P}+\mu \biggr)^{-1} \biggr]-\frac {r\widetilde{S}}{2K} \biggr\} u^{2} \\ &\qquad{}+ \biggl[\frac{K}{2r\widetilde{S}} \biggl(r-\frac{3r}{K}\widetilde{S}- \alpha \widetilde{P}-\beta\widetilde{P}-\mu \biggr)^{2} + \frac{\eta_{2}}{\delta} \biggl(\frac{r}{K}\widetilde{S}+2\beta\widetilde {P}+ \mu+\delta \biggr)^{2}\\ &\qquad{}- \biggl(\frac{r}{K}\widetilde{S}+\beta \widetilde{P}+\mu \biggr) \biggr]I^{2} \\ &\qquad{}-\frac{\eta_{2}\delta}{2\theta^{2}}w^{2}- \biggl(\frac{\delta}{\theta}+\eta _{1}\theta\beta\widetilde{P} \biggr)Iw+M_{1}(u,I,w)\\ &\qquad{}+ \eta_{2} \biggl(u+I+\frac {1}{\theta}w \biggr) \biggl(- \frac{r}{K}u^{2}-\frac{r}{K}uI-2\beta Iw \biggr). \end{aligned}$$

Let

$$\eta_{2}=\frac{r\widetilde{S}}{4K} \biggl[\alpha\widetilde{P}+ \frac{1}{\delta } \biggl(r-\frac{2r}{K}\widetilde{S}-\delta \biggr)^{2} +\frac{1}{4} \biggl(r-\frac{3r}{K} \widetilde{S}-2\beta\widetilde{P}-\mu \biggr)^{2} \biggl( \frac{r}{K}\widetilde{S}+2\beta\widetilde{P}+\mu \biggr)^{-1} \biggr]^{-1}, $$

so that

$$\eta_{2} \biggl[\alpha\widetilde{P}+\frac{1}{\delta} \biggl(r- \frac{2r}{K}\widetilde {S}-\delta \biggr)^{2} + \frac{1}{4} \biggl(r-\frac{3r}{K}\widetilde{S}-2\beta\widetilde{P}- \mu \biggr)^{2} \biggl(\frac{r}{K}\widetilde{S}+2\beta \widetilde{P}+ \mu \biggr)^{-1} \biggr]=\frac {r\widetilde{S}}{4K}. $$

Then we get

$$\begin{aligned} &\eta_{2}LV_{1}+LV_{2}+\eta_{1}LV_{4} \\ &\quad\leq-\frac{r\widetilde{S}}{4K}u^{2}-\frac{\eta_{2}\delta}{4\theta^{2}}w^{2} + \biggl[\frac{K}{2r\widetilde{S}} \biggl(r-\frac{3r}{K}\widetilde{S}-\alpha \widetilde{P}-\beta\widetilde{P}-\mu \biggr)^{2}+\frac{\eta_{2}}{\delta} \biggl(\frac {r}{K}\widetilde{S}+2\beta\widetilde{P}+\mu+\delta \biggr)^{2} \\ &\qquad{}+\frac{\theta^{2}(\frac{\delta}{\theta} +\eta_{1}\theta\beta\widetilde{P})^{2}}{\delta\eta_{2}} - \biggl(\frac{r}{K}\widetilde{S}+\beta \widetilde{P}+\mu \biggr) \biggr]I^{2}+M_{1}(u,I,w) \\ &\qquad{}+\eta_{2} \biggl(u+I+\frac{1}{\theta}w \biggr) \biggl(- \frac{r}{K}u^{2}-\frac {r}{K}uI-2\beta Iw \biggr). \end{aligned}$$

Finally, we obtain

$$\begin{aligned} LV={}&\eta_{2}LV_{1}+LV_{2}+\eta_{1}LV_{4}+ \eta_{3}LV_{3} \\ \leq{}&{-}\frac{r\widetilde{S}}{4K}u^{2}-\frac{\eta_{2}\delta}{4\theta^{2}}w^{2} + \biggl[\frac{K}{2r\widetilde{S}} \biggl(r-\frac{3r}{K}\widetilde{S}-\alpha \widetilde{P}-\beta\widetilde{P}-\mu \biggr)^{2}\\ &{}+\frac{\eta_{2}}{\delta} \biggl(\frac {r}{K}\widetilde{S}+2\beta\widetilde{P}+\mu+\delta \biggr)^{2} \\ &{}+\frac{\theta^{2}(\frac{\delta}{\theta} +\eta_{1}\theta\beta\widetilde{P})^{2}}{\delta\eta_{2}} - \biggl(\frac{r}{K}\widetilde{S}+\beta \widetilde{P}+\mu \biggr)-\eta_{3} \biggl(\beta \widetilde{P}+\mu- \lambda \widetilde{S}-\frac{1}{2}\sigma^{2}\widetilde {S}^{2} \biggr) \biggr]I^{2}\\ &{}+M_{1}(u,I,w) \\ &{}+\eta_{2} \biggl(u+I+\frac{1}{\theta}w \biggr) \biggl(- \frac{r}{K}u^{2}-\frac {r}{K}uI-2\beta Iw \biggr)+ \eta_{3} \biggl(\lambda u-\beta w+\frac{1}{2}\sigma ^{2}u^{2}+\sigma^{2}\widetilde{S}u \biggr)I^{2}. \end{aligned}$$

Put

$$\begin{aligned} \eta_{3}={}& \biggl[\frac{K}{2r\widetilde{S}} \biggl(r-\frac{3r}{K} \widetilde{S}-\alpha \widetilde{P}-\beta\widetilde{P}-\mu \biggr)^{2}+ \frac{\eta_{2}}{\delta} \biggl(\frac {r}{K}\widetilde{S}+2\beta\widetilde{P}+ \mu+ \delta \biggr)^{2}+\frac{\theta ^{2}(\frac{\delta}{\theta} +\eta_{1}\theta\beta\widetilde{P})^{2}}{\delta\eta_{2}} \biggr] \\ &{}\times \biggl(\beta\widetilde{P}+\mu-\lambda\widetilde{S}-\frac{1}{2} \sigma ^{2}\widetilde{S}^{2} \biggr)^{-1}, \end{aligned}$$

so that

$$\begin{aligned} &\frac{K}{2r\widetilde{S}} \biggl(r-\frac{3r}{K}\widetilde{S}-\alpha\widetilde {P}-\beta\widetilde{P}-\mu \biggr)^{2}+\frac{\eta_{2}}{\delta} \biggl( \frac {r}{K}\widetilde{S}+2\beta\widetilde{P}+\mu+\delta \biggr)^{2}+\frac{\theta ^{2}(\frac{\delta}{\theta} +\eta_{1}\theta\beta\widetilde{P})^{2}}{\delta\eta_{2}} \\ &\quad{}-\eta_{3} \biggl(\beta\widetilde{P}+\mu-\lambda\widetilde{S}- \frac{1}{2}\sigma ^{2}\widetilde{S}^{2} \biggr)=0. \end{aligned}$$

Then we get

$$\begin{aligned} LV={}&\eta_{2}LV_{1}+LV_{2}+\eta_{1}LV_{4}+ \eta_{3}LV_{3} \\ \leq{}&- \frac{r\widetilde{S}}{4K}u^{2}- \biggl(\frac{r}{K} \widetilde{S}+\beta\widetilde {P}+\mu \biggr)I^{2}-\frac{\eta_{2}\delta}{4\theta^{2}}w^{2} +M_{1}(u,I,w) \\ &{}+\eta_{2} \biggl(u+I+\frac{1}{\theta}w \biggr) \biggl(- \frac{r}{K}u^{2}-\frac {r}{K}uI-2\beta Iw \biggr)+ \eta_{3} \biggl(\lambda u-\beta w+\frac{1}{2}\sigma ^{2}u^{2}+\sigma^{2}\widetilde{S}u \biggr)I^{2}. \end{aligned}$$

Let $\widetilde{\lambda}=\min\{\frac{r\widetilde{S}}{4K},\frac {r}{K}\widetilde{S}+\beta\widetilde{P}+\mu,\frac{\eta_{2}\delta}{4\theta ^{2}} \}$. Then

$$LV\leq-\widetilde{\lambda} \bigl|x(t) \bigr|^{2}+o \bigl(\bigl|x(t)\bigr|^{2} \bigr). $$

Hence, $LV(x)$ is negative-definite in a sufficiently small neighborhood of $x = 0$ for $t\geq0$. From Lemma A.3 of Mao [17] we therefore conclude that the zero solution of (6.1) is stochastically asymptotically stable. □

## Numerical simulations

In this section, we make numerical simulations to illustrate our results by using Milstein’s higher-order method [18]. Variables and parameters used in the models of susceptible prey-infected prey-predator population interaction are given by Chattopadhyay *et al.* [11], Table 2, where

$$r=3,\qquad K=45,\qquad \beta=0.05,\qquad \mu=0.24,\qquad \theta=0.4, \qquad \delta=0.09. $$

First, we take $\alpha=0.004$, $\lambda=0.003$, $\sigma=0.045$. In this case,

$$\alpha=0.004< \frac{\delta}{K\theta}=0.005, \qquad\sigma^{2}=0.002025< \frac {2(r+K\lambda)(\mu-K\lambda)}{\lambda K^{3}}=0.002408. $$

**Figure 1:**
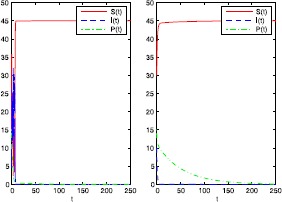
**Numerical simulation of the solution of system (** **1.2** **) and its corresponding deterministic system (** **1.1** **) with** $\pmb{\alpha=0.004}$ **,** $\pmb{\lambda=0.003}$ **,** $\pmb{\sigma=0.045}$ **and with the initial values** $\pmb{S(0)=30}$ **,** $\pmb{I(0)=10}$ **,** $\pmb{P(0)=15}$ **.**

We can therefore conclude by Theorem 4.1 that the equilibrium $E_{1}=(45,0,0)$ of system (1.2) is stochastically asymptotically stable in the large. The numerical simulations in Figure 1 support these results clearly.

Noting that

$$\sigma^{2}=0.002025>\max \biggl\{ \frac{\lambda}{K}=0.000067, \frac{\lambda ^{2}}{2\mu}=0.00001875 \biggr\} =0.000067 $$

and

$$\alpha=0.004< \frac{\delta}{K\theta}=0.005, $$

we see that conditions (a) and (c) of Theorem 4.2 are satisfied. Therefore, by Theorem 4.2, for the initial values $S(0)=30$, $I(0)=10$, and $P(0)=15$, the solution of system (1.2) obeys

$$\begin{aligned} &\limsup_{t\rightarrow\infty}\frac{\log I(t)}{t}\leq-0.2378< 0 \quad \mbox{a.s.}, \\ &\limsup_{t\rightarrow\infty}\frac{\log P(t)}{t}\leq-0.018< 0\quad \mbox{a.s.}, \\ &\lim_{t\rightarrow\infty}\frac{1}{t} \int^{t}_{0}S(s)\,ds=45 \quad\mbox{a.s.} \end{aligned}$$

The numerical simulations in Figure 1 support these results clearly, illustrating extinction of the infected prey and the predator.

**Figure 2:**
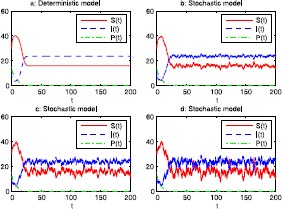
**Numerical simulation of the solution of system (** **1.2** **) and its corresponding deterministic system (** **1.1** **) with initial value** $\pmb{S(0)=30}$ **,** $\pmb{I(0)=10}$ **,** $\pmb{P(0)=15}$ **: (a) is with** $\pmb{\alpha=0.004}$ **,** $\pmb{\lambda=0.015}$ **; (b) with** $\pmb{\alpha =0.004}$ **,** $\pmb{\lambda=0.015}$ **,** $\pmb{\sigma=0.002}$ **; (c) with** $\pmb{\alpha=0.004}$ **,** $\pmb{\lambda =0.015}$ **,** $\pmb{\sigma=0.004}$ **; (d) with** $\pmb{\alpha=0.004}$ **,** $\pmb{\lambda=0.015}$ **,** $\pmb{\sigma=0.006}$ **.**

Next, we choose $\alpha=0.004$ and $\lambda=0.015$. Then

$$\overline{S}=\frac{\mu}{\lambda}=16,\qquad \overline{I}=\frac{r(1-\frac {\overline{S}}{K})}{\frac{r}{K}+\lambda}=23.67, $$

and the conditions

$$\mu=0.24 < \lambda K=0.675, \qquad \alpha=0.004< \frac{\delta}{K \theta}=0.005 $$

are satisfied. Therefore, by Theorem 5.1, $P(t)$ tends to zero exponentially with probability one. We see that the difference between the solution of system (1.2) and $E_{2}=(16, 23.67, 0)$ in time average is related to the intensity of the white noise. The weaker the white noise, the smaller the difference. The numerical simulations in Figure 2 support these results clearly, illustrating that the solution of system (1.2) is surrounding $E_{2}$ randomly oscillating, and the extent vibrating enhances gradually with gradual increase of *σ*.

Finally, we take $\alpha=0.3$, $\lambda=0.008$, $\sigma=0.2$. In this case, we compute

$$\begin{aligned}& \widetilde{S}=\frac{\delta}{\theta\alpha}=0.75, \qquad\widetilde{P}=\frac {r}{\alpha} \biggl(1-\frac{\widetilde{S}}{K} \biggr)=9.833, \\& \frac{\delta\lambda}{\mu\theta}=0.0075< \alpha=0.3, \qquad\frac{\mu }{K}=0.0053< \lambda=0.008, \\& \sigma^{2}=0.04< \frac{2(\beta\widetilde{P}+\mu-\lambda\widetilde {S})}{\widetilde{S}^{2}}=2.58. \end{aligned}$$

**Figure 3:**
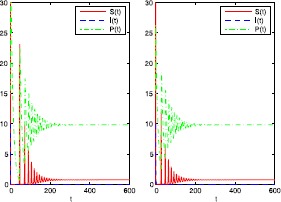
**Numerical simulation of the solution of system (** **1.2** **) and its corresponding deterministic system (** **1.1** **) with** $\pmb{\alpha=0.3}$ **,** $\pmb{\lambda=0.008}$ **,** $\pmb{\sigma=0.2}$ **and with initial values** $\pmb{S(0)=30}$ **,** $\pmb{I(0)=10}$ **,** $\pmb{P(0)=15}$ **.**

We can therefore conclude, by Theorem 6.1, that the equilibrium $E_{3}=(0.75,0,9.833)$ of system (1.2) is stochastically asymptotically stable. The numerical simulations in Figure 3 support these results clearly.

## Conclusion

In this paper, we have proposed and analyzed an ecoepidemiological model with linear mass-action functional response perturbed by white noise. Based on this model, we mainly have showed that system (1.2) has a unique positive global solution and investigated how the four equilibria $E_{0}$, $E_{1}$, $E_{2}$, and $E_{3}$ of system (1.1) will be under stochastic perturbation. The key parameters are one ecological parameter *α*, predators’ attack rate on susceptible prey, and one epidemiological parameter *λ*, the rate of infection.

(i) System (1.1) is unstable around $E_{0}$ for all parametric values. We show that the equilibrium $E_{0}$ of system (1.2) is stochastically unstable in Theorem 3.1.

(ii) If $\lambda< \frac{\mu}{K} $ and $\alpha<\frac{\delta}{K\theta}$, then system (1.1) is globally asymptotically stable around the equilibrium $E_{1}$. Theorem 4.1 shows that if $K \lambda<\mu-\frac{\lambda K^{3}\sigma ^{2}}{2(r+K\lambda)}$ and $\alpha<\frac{\delta}{K\theta}$, then the equilibrium $E_{1}$ of system (1.2) is stochastically asymptotically stable in the large. Theorem 4.2 shows that, under some conditions, the disease will die out, the predator population will go into extinction, and the prey population will approach the carrying capacity *K*. Biologically, it implies that if both the infection rate and the search rate of susceptible prey are low, then the infected prey and predator population cannot survive, and the system converges to the equilibrium where only healthy prey exists.

(iii) If $\lambda>\frac{\mu}{K}$, then the equilibrium $E_{2}$ of system (1.1) exists. Theorem 5.1 shows that the difference between the solution of system (1.2) and $E_{2}$ in time average is only relation with the intensity of the white noise. The weaker the white noise, the smaller the difference. So there is approximate stability, provided that *σ* is sufficiently small. Biologically, this implies that if the infection rate is too high and the search rate of susceptible population is moderate, then the predator population cannot survive, and the system converges to the equilibrium where susceptible prey and infected prey coexist in the form of a stable equilibrium.

(iv) If $\lambda> \frac{\mu}{K} $ and $\alpha>\frac{\delta\lambda}{\mu \theta}$, then system (1.1) is globally asymptotically stable around the equilibrium $E_{3}$. We also show (Theorem 6.1) that if $\lambda> \frac{\mu}{K}$ and $\alpha>\frac{\delta\lambda}{\mu\theta}$, then, under certain conditions, the equilibrium $E_{3}$ of system (1.2) is stochastically asymptotically stable. Biologically, it implies that in case of higher infection rate and higher predation rate, all trajectories with the default values converge to the disease-free equilibrium $E_{3}$, where susceptible prey and predator population coexist in the form of a stable equilibrium.

## Appendix 1

In this section, we list some definitions and theory used in the previous sections.

In general, consider a *d*-dimensional stochastic differential equation

A.1 $$ dx(t)=f \bigl(x(t),t \bigr)\,dt+g \bigl(x(t),t \bigr)\,dB(t) \quad \mbox{for } t \geq t_{0}.$$

Assume that $f(0,t)=0$ and $g(0,t)=0$ for all $t\geq t_{0}$. So $x(t)\equiv0$ is a solution of equation (A.1), called the trivial solution or equilibrium position.

### Definition A.1

([17])

(i) The trivial solution of system (A.1) is said to be stochastically stable or stable in probability if for every pair of $\varepsilon\in(0, 1)$ and $r > 0$, there exists $\delta= \delta(\varepsilon, r, t_{0})>0$ such that

$$P \bigl\{ \bigl|x(t; t_{0}, x_{0})\bigr|< r \mbox{ for all } t \geq t_{0} \bigr\} \geq 1- \varepsilon $$

whenever $|x_{0}| < \delta$. Otherwise, it is said to be stochastically unstable.

(ii) The trivial solution is said to be stochastically asymptotically stable if it is stochastically stable; moreover, for every $\varepsilon\in(0, 1)$, there exists $\delta_{0}= \delta _{0}(\varepsilon, t_{0})>0$ such that

$$P \Bigl\{ \lim_{t\rightarrow\infty}x(t; t_{0}, x_{0})=0 \Bigr\} \geq1- \varepsilon $$

whenever $|x_{0}| < \delta_{0}$.

(iii) The trivial solution is said to be stochastically asymptotically stable in the large if it is stochastically asymptotically stable; moreover, for all $x_{0} \in R^{d}$,

$$P \Bigl\{ \lim_{t\rightarrow\infty}x(t; t_{0}, x_{0})=0 \Bigr\} = 1. $$

### Lemma A.1

(Strong law of large numbers [17])

*Let*$M =\{M_{t}\}_{t\geq0 }$*be a real*-*valued continuous local martingale vanishing at*$t = 0$. *Then*

$$\lim_{t\rightarrow\infty}\langle M,M \rangle_{t}=\infty \quad \textit{a.s.} \quad\Rightarrow\quad\lim_{t\rightarrow\infty} \frac{M_{t}}{\langle M,M \rangle _{t}}=0 \quad\textit{a.s. } $$

*and also*

$$\limsup_{t\rightarrow\infty}\frac{\langle M,M \rangle_{t}}{t}< \infty \quad\textit{a.s.} \quad\Rightarrow\quad\lim_{t\rightarrow\infty}\frac{M_{t}}{t}=0\quad \textit{a.s.} $$

### Lemma A.2

([17])

*If there exists a positive*-*definite decreasing radially unbounded function*$V(x,t) \in C^{2,1}(R^{d} \times[t_{0},\infty];R_{+})$*such that*$LV(x,t)$*is negative*-*definite*, *then the trivial solution of equation* (A.1) *is stochastically asymptotically stable in the large*.

### Lemma A.3

([17])

*If there exists a positive*-*definite decreasing function*$V(x,t) \in C^{2,1}(S_{h} \times[t_{0},\infty];R_{+})$*such that*$LV(x,t)$*is negative*-*definite*, *then the trivial solution of system* (A.1) *is stochastically asymptotically stable*.

## Appendix 2: The rest of the proof of Theorem 2.1

Let $\widetilde{K}=\mu+\frac{\delta}{\theta}+\max\{r+\frac{r}{K},\frac {r}{K}+\lambda+\beta,\theta(\alpha+\beta)\}\frac{B}{\eta}+K^{2}\sigma^{2}$. Then

$$\int_{0}^{\tau_{k}\wedge T}\,dV \bigl(S(t),I(t),P(t) \bigr)\leq \int_{0}^{\tau _{k}\wedge T}\widetilde{K}\,dt+ \int_{0}^{\tau_{k}\wedge T}\sigma \bigl(I(t)-S(t) \bigr)\,dB(t). $$

Taking expectations yields

2.2 $$\begin{aligned} &E \bigl[V \bigl(S(\tau_{k}\wedge T),I( \tau_{k}\wedge T),P(\tau_{k}\wedge T) \bigr) \bigr] \\ &\quad\leq V \bigl(S(0),I(0),P(0) \bigr)+E \int_{0}^{\tau_{k}\wedge T}\widetilde{K}\,dt \\ &\quad\leq V \bigl(S(0),I(0),P(0) \bigr)+\widetilde{K}T. \end{aligned}$$

Set $\Omega_{k}=\{\tau_{k}\leq T \}$ for $k \geq k_{1}$. Then, by (2.1), $\mathbb{P}(\Omega_{k})\geq\varepsilon$. Note that, for every $\omega \in\Omega_{k}$, at least one of $S(\tau_{k},\omega)$, $I(\tau_{k},\omega)$, and $P(\tau_{k},\omega )$ equals either *k* or $\frac{1}{k}$, and hence $V(S(\tau_{k}\wedge T),I(\tau_{k}\wedge T),P(\tau_{k}\wedge T))$ is no less than either

$$k-1-\log k $$

or

$$\frac{1}{k}-1-\log\frac{1}{k}=\frac{1}{k}-1+\log k. $$

Consequently,

$$V \bigl(S(\tau_{k}\wedge T),I(\tau_{k}\wedge T),P( \tau_{k}\wedge T) \bigr) \geq(k-1-\log k)\wedge \biggl( \frac{1}{k}-1+\log k \biggr). $$

It then follows from (2.1) and (2.2) that

$$\begin{aligned} &V \bigl(S(0),I(0),P(0) \bigr)+\widetilde{K}T \\ &\quad\geq E \bigl[1_{\Omega_{k}(\omega)}V \bigl(S(\tau_{k}\wedge T),I( \tau_{k}\wedge T),P(\tau_{k}\wedge T) \bigr) \bigr] \\ &\quad\geq\varepsilon \biggl[(k-1-\log k)\wedge \biggl(\frac{1}{k}-1+\log k \biggr) \biggr], \end{aligned}$$

where $1_{\Omega_{k}(\omega)}$ is the indicator function of $\Omega _{k}(\omega)$. Letting $k\rightarrow\infty$ leads to the contradiction $\infty>V(S(0),I(0),P(0))+\widetilde{K}T=\infty$. So we have $\tau _{\infty}=\infty$ a.s. This completes the proof of Theorem 2.1.
